# An ancient history of gene duplications, fusions and losses in the evolution of APOBEC3 mutators in mammals

**DOI:** 10.1186/1471-2148-12-71

**Published:** 2012-05-28

**Authors:** Carsten Münk, Anouk Willemsen, Ignacio G Bravo

**Affiliations:** 1 Clinic for Gastroenterology, Hepatology and Infectiology, Medical Faculty, Heinrich-Heine-University, Düsseldorf, Germany; 2 Genomics and Health, Centre for Public Health Research (CSISP), Valencia, Spain; 3 Infections and Cancer, Catalan Institute of Oncology (ICO) | Bellvitge Institute of Biomedical Research (IDIBELL), Barcelona, Spain; 4 Infections and Cancer, Catalan Institute of Oncology (ICO), Avda. Gran Via, 199-203, L’Hospitalet de Llobregat, Barcelona, 08908, Spain

**Keywords:** APOBEC, Cytydine deaminase, Gene duplication, Subfunctionalisation, Virus/host arms race

## Abstract

**Background:**

The APOBEC3 (A3) genes play a key role in innate antiviral defense in mammals by introducing directed mutations in the DNA. The human genome encodes for seven A3 genes, with multiple splice alternatives. Different A3 proteins display different substrate specificity, but the very basic question on how discerning self from non-self still remains unresolved. Further, the expression of A3 activity/ies shapes the way both viral and host genomes evolve.

**Results:**

We present here a detailed temporal analysis of the origin and expansion of the A3 repertoire in mammals. Our data support an evolutionary scenario where the genome of the mammalian ancestor encoded for at least one ancestral A3 gene, and where the genome of the ancestor of placental mammals (and possibly of the ancestor of all mammals) already encoded for an A3Z1-A3Z2-A3Z3 arrangement. Duplication events of the A3 genes have occurred independently in different lineages: humans, cats and horses. In all of them, gene duplication has resulted in changes in enzyme activity and/or substrate specificity, in a paradigmatic example of convergent adaptive evolution at the genomic level. Finally, our results show that evolutionary rates for the three A3Z1, A3Z2 and A3Z3 motifs have significantly decreased in the last 100 Mya. The analysis constitutes a textbook example of the evolution of a gene locus by duplication and sub/neofunctionalization in the context of virus-host arms race.

**Conclusions:**

Our results provide a time framework for identifying ancestral and derived genomic arrangements in the APOBEC loci, and to date the expansion of this gene family for different lineages through time, as a response to changes in viral/retroviral/retrotransposon pressure.

## Background

Cytidine deaminases of the APOBEC3 (A3) gene family have a broad antiviral activity against retroviruses, can inhibit LTR- and non-LTR-retrotransposons, parvoviruses, hepadnaviruses, flaviviruses and paramyxoviruses, and might repress also TT-viruses, papillomaviruses and herpesviruses [1-9]. Under the already tested additional viruses, adenoviruses, poxviruses and influenza viruses replicate well irrespective of A3s [3,10,11]. The hunt is on for the identification of additional viral targets [12]. The APOBEC3 gene family encodes a characteristic zinc (Z)-coordinating catalytic motif (His-X-Glu-X_23–28_-Pro-Cys-*X*_2–4_-Cys) [13] and the A3 proteins can be classified according to the presence of an A3Z1, A3Z2 or A3Z3 motif [14-16]. A3s act by deaminating cytidine into uridine (EC 3.5.4.5) using single-stranded DNA as a substrate. This DNA editing results in the introduction of mutations that eventually render the target genome inactive. However, uncontrolled chemical editing of DNA sequences puts at risk the genomic information in the cell [17,18] and many mechanistic questions regarding A3 activity and substrate specificity remain open.

During retroviral budding, A3 molecules incorporated into progeny retroviral particles are carried over within the virion and counteract the new infection upon release in the cytoplasm of the newly infected cell. Retroviruses have evolved mechanisms to prevent encapsidation of A3s into viral particles. The Vif protein in lentiviruses, the Bet protein in foamyviruses and the nucleocapsid protein in *Human T-cell lymphotrophic virus* accomplish this anti-antiviral activity [19-24]. The expression of A3s is not restricted to the immune system [25], and different cell types may express different A3 repertoires, and even different variants may present different subcellular location [26]. Thus, some retroviruses without *vif* or *bet* genes might escape A3-mediated antiviral inhibition by a restriction of their cellular tropisms, as it is discussed for the *Equine infectious anemia virus*[27]. Several aspects on how retroviruses cope with the deaminase activity of the A3s encoded by their host species are still a matter of debate [28,29]. Thus, and despite several studies, it remains unknown which strategies the *Moloney murine leukemia virus* or the *Mouse mammary tumor virus* have evolved to prevent inhibition by the murine A3 [30-36].

In mammals the A3 locus appears always flanked by the CBX6 and CBX7 genes but there is ample variation across species, regarding the number and arrangement of A3 individual genes, presence of fused genes, expression pattern, splice alternatives and read-through mechanisms and substrate specificity, even in related species. Thus, although Primates and Rodents are relatives and belong together within Euarchontoglires, the human genome contains seven A3 genes, four of them resulting from the fusion of two A3 domains, while the mouse genome encodes for a single A3Z2-A3Z3 fused gene [37]. Additionally the dog genome presents two A3 genes, while there are four A3 genes in cats, two to three in pigs, sheep and cow and six in horses [15,16,27], and all these species belong together within Laurasiatheria. The finding of A3Z1, A3Z2, and A3Z3 genes in these two mammalian lineages strongly indicates that their ancestor (Boreoeutheria) was probably equipped with a single copy of each gene.

One important mechanism of genome evolution and for the appearance of novel gene functions is gene duplication [38]. Genes within a genome that are descendants of gene duplications are paralogs, while two genes in different species that derive from a single gene in the last common ancestor of both species are orthologs. Paralogs derive either from an ancestral duplication (“outparalogs”) or they derive from a lineage-specific duplication (“inparalogs”), giving rise to co-orthologous relationships [39]. Several models for the emergence, maintenance and evolution of gene copies have been proposed (for a review see [40]). The duplication of genes may in some cases have no immediate consequence for the host, but in other cases can be deleterious or linked to disease [41], or confer an selective advantage [42]. For the antiviral A3 genes evolutionary solutions reflect the trade off between a potential self-toxicity against cellular DNA -in cases of an A3 exacerbated response- and the emergence of viral pathogens if low A3 activity and/or diversity allow for restriction escape variants. Fixation of duplicated A3 genes and the subsequent preservation in certain population is likely driven by a strong selective advantage for the individuals carrying additional copies of the gene/s. Any subsequent acquisition of genetic differences between the gene copies can alter the chances of both copies being preserved and might change the function of the encoded proteins and result either in gene loss, ‘neo-functionalization’ [38] or ‘sub-functionalization’ [43].

We present here a time scale of the evolution of the A3 loci, arising from their common origin with other cytidine deaminases. According to our results, the track of duplications in the A3 locus started with the ancestral gene itself. The appearance of the three clades within the A3 family dates back to emergence of placental mammals. The evolutionary history of A3s has since then been landmarked by duplication events especially in Primates, but also in Perissodactyla and Carnivora, as well as by deletions events, such as in Rodentia. Three main trends can be observed: first, a sustained decrease in evolutionary rate for the A3 subfamilies in the last 100 Mya; second, duplication events have occurred in the A3Z1 and A3Z2 subfamilies, but not in the A3Z3; third, duplication events accumulate in the last 50 Mya. The molecular mechanisms that generate the tandem duplication of A3, and the evolutionary pressures that drive the sub/neofunctionalisation and eventually selection of the duplicated genes still need to be identified.

## Results

### There are three main clades of A3 genes

**Figure 1:**
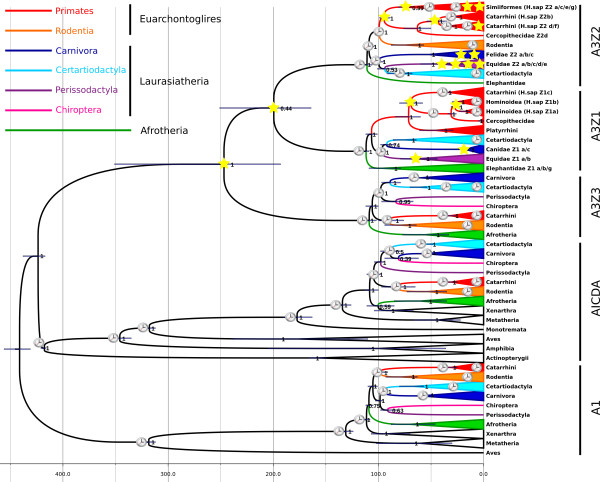
**Dated Bayesian phylogenetic tree for the A3 family using A1 sequences as outgroup.** Scale bar given in Mya. Values at the nodes correspond to ML bootstrap support. Error bars encompass 95% HPD for the age of the nodes. Star symbols indicate duplication events in the corresponding lineage and moment in time. Clock cartoons labels the nodes used for calibration. Mammalian clades are labelled using colours (code in the inset). No sequences from Xenarthra could be found in our searches.

We have found sequences corresponding to A3 genes in extant members of Laurasiatheria (seven Carnivora, seven Cetartiodactyla, one Perissodactyla and one Chiroptera species), Euarchontoglires (three Rodentia and seventeen Primates species) and Afrotheria (one Hyracoidea and one Proboscidea species). However, BLAST and PSI-BLAST searches of genomic, EST and other sequence databases failed to find A3 in any other taxon within other placental mammals (e.g. Xenarthra) marsupials, monotremes, or in non-mammals. A selection of 34 AICDA sequences (23 Eutheria species, three Metatheria, one Prototheria, two Aves, two Amphibia and three Actinopterygii species) was identified as sister taxa to all A3s, while a selection of 24 A1 sequences (21 Eutheria species, two Metatheria and one Aves species) was identified as outgroup. An exhaustive list of species and accession numbers is given in Additional file 1: Table S1. Using this dataset, we performed first phylogenetic reconstruction using both maximum likelihood and Bayesian inference, identified local topologies suitable for molecular dating, and then performed time inference introducing this information as temporal constrains using a relaxed clock approach. The global results are shown in Figure 1. More detailed results are shown in Additional file 2: Figure S1 which shows the bootstrap values of the ML analysis and Additional file 3: Figure S2 which contains the 95% highest posterior density (HPD) of the node ages.

The most recent common ancestor (MRCA) of the A1s, A3s and AICDA sequences can be dated 441 Mya (95% HPD, 431–456 Mya). The MRCA of the A3s and AICDA sequences can be dated 424 Mya (95% HPD, 417–438 Mya), while the MRCA of extant A3 sequences can be dated 247 Mya (95% HPD 193–351 Mya) (Figure 1). The three members of the A3 family radiated later at comparable time points: 112 Mya (95% HPD 109–113 Mya) for A3Z1; 111 Mya (95% HPD 106–113 Mya) for A3Z2; and 109 Mya (95% HPD 102–113 Mya) for A3Z3. The relationships among the three A3 subfamilies could not be solved with certainty, but both ML (44% bootstrap support) and Bayesian inference (0.52 Bayesian posterior probability) suggested a sisterhood relationship between A3Z1 and A3Z2, with a MRCA dated 199 Mya (95% HPD 164–251 Mya). Only more quality data from Afrotheria, or the discovery of A3 genes in Monotremes, Marsupials and/or Xenarthra may help us define and date these basal nodes with confidence.

### Evolution of the A3 loci in Euarchontoglires

**Figure 2:**
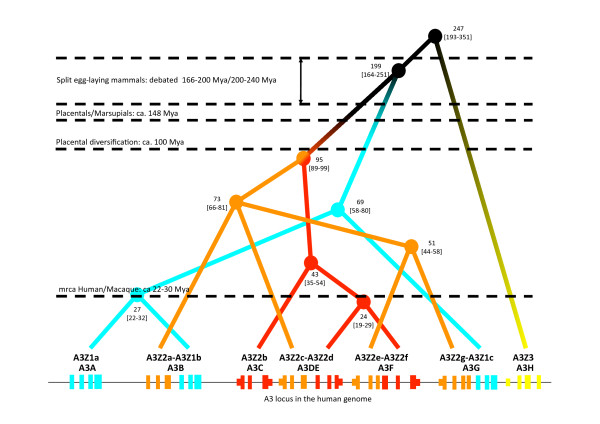
**Projection of the A3 phylogenetic relationships onto the human genomic locus.** Exon composition and gene arrangement of the seven A3 genes are displayed. Labels indicate median age [95% HPD] of the corresponding nodes. Several milestones in the evolution of mammals are indicated.

Rodents and Primates are the two main orders in Euarchontoglires. We could identify different A3 genes in the genomes of platyrrhines (New World monkeys) and catarrhines (Old World monkeys and apes). In modern humans eleven A3 open reading frames exist, forming seven genes encoding a single Z domain or a Z-Z domain, either fused A3Z2-A3Z2 or A3Z2-A3Z1 (Figure 2). This is by far the most complex known organisation of the A3 loci. We must however keep in mind that our knowledge on the structure of the A3 loci in other primate genomes, especially regarding transcriptomic data, is still fragmentary.

#### A3Z1 genes in primates

The A3Z1 gene appears in humans in three copies, named Z1a, Z1b and Z1c, and we will name the corresponding orthologs according to the nomenclature in humans (Figure 2). The MRCA of A3Z1 genes can be dated 71 Mya (95% HPD 60–84), which grossly corresponds to the basal divergence time of primates, some 73–87 Mya. Orthologs of the A3Z1 genes can be found in the genomes of several Haplorrhini species, including Catarrhini (Old World monkeys and apes) and Platyrrhini (New World monkeys). We could only detect one A3Z1 gene in the platyrrhines genomes we have browsed, and these sequences are the outgroup of the Z1a, Z1b and Z1c found in catarrhines. Phylogenetic relationships and inferred divergence times among the Z1c orthologs grossly correspond to those among the corresponding species. Regarding the Z1a and Z1b genes, we could identify the orthologs of both human genes in the genomes of the common chimpanzee, western gorilla, northern white-cheeked gibbon and Rhesus macaque. The ortholog of the Z1b human gene could be identified in the Sumatran orangutang. The duplication event that generated the Z1a and Z1b genes can be dated some 27 Mya (95% HPD 22–32). This timing could be compatible with the Hylobatidae/Hominidae split some 18–24 Mya, although it matches well the Cercopithecidae/Hominoidea split some 26–34 Mya [44].

A plausible scenario for the evolution of the A3Z1 loci in primates would include a basal split, generating the ancestors of the Z1a/Z1b (hereafter Z1ab) and the Z1c genes. Whether this first duplication event predates the early split Haplorrhini (tarsiers, monkeys and apes)/Strepsirrhini (non-tarsier prosimians) or is rather exclusive to the Haplorrhini lineage is not clear and must still be solved, since there are no sequence data available for Strepsirrhini. In Catarrhini, the ancestral Z1c gene may have evolved without further duplication event. The ancestral Z1ab gene underwent duplication and generated the ancestral Z1a and Z1b genes. Orthologs of both Z1a and Z1b are present in all hominids [45], as the MRCA to both genes predates well the split Ponginae/Homininae. Future identification of Z1ab genes in hylobatids and in cercopithecoids will clear whether the Z1a/Z1b duplication event is basal to catarrhini or to Hominoidea.

#### A3Z2 genes in primates

Our fragmentary information on the A3 gene content makes it impossible to reconstruct with confidence the evolutionary relationships among the A3Z2 genes in primates. Additionally, a number of A3Z2 sequences arise from genomic material while others belong to cDNA, and the complex alternative splicing of the A3 genes may disturb further the algorithms for phylogenetic inference. Finally, in this locus gene conversion may have played a key role, given the large number of gene duplication events in this A3 subfamilly -up to six in humans- and the nature of the locus, where the copies generated remain in a tandem arrangement. A proper phylogenetic reconstruction will thence need to wait until genomic sequences with better coverage are available for a larger number of primate species, especially if encompassing Strepsirrhini and Tarsiidae. A basal split event some 94 Mya (95% HPD 89–99) generated the two main lineages Z2bdf and Z2aceg. This timing could match the basal strepsirrhini/haplorrhini diversification time in primates (some 87 Mya), although it would be more compatible with one duplication exclusive to haplorrhini (MRCA some 85 Mya) or even to simiiformes. In either case our interpretation implies that the ancestral simiiformes already carried two copies of the A3Z2 genes in their genomes. The Z2bdf group comprises exclusively sequences from catarrhines, with an estimated MRCA around 43 Mya (95% HPD 35–54). Sequences here cluster into two groups: Z2b and Z2df. Both clusters contain sequences from cercopithecoids and from hominoids. It can thus be inferred that the duplication event that generated both groups took place before the divergence between cercopithecoids and hominoids some 29 Mya. This timing is compatible with the estimated MRCAs around 28 Mya (95% HPD 23–34) for Z2b and 32 Mya (95% HPD 28–34) for Z2df. Within the Z2aceg group, phylogenetic inference cannot solve the fine relationships. Only a major, monophyletic group containing the Z2g genes can be clearly defined, containing sequences of both catarrhines and platyrrhines. Phylogenetic relationships among Z2g genes grossly correspond to those of the corresponding species: platyrrhines are basal to the cluster, with calculated split time 39 Mya (95% HPD 34–45), and cercopithecoids and hominids cluster separately, with a MRCA around 24 Mya (95% HPD 23–28).

A plausible scenario for the evolution of the A3Z2 genes in primates would include a first basal split Z2aceg/Z2bdf, previous to the split between platyrrhini and catarrhini. The ancestral Z2aceg underwent a second duplication Z2ace/Z2g also before this split event. Modern Z2a, Z2c and Z2e human genes appeared after subsequent duplication events. On the other hand, the ancestral Z2bdf underwent a first duplication Z2b/Z2df at least before the divergence between cercopithecoids and hominoids. The basal position of human Z2d with respect to Z2f sequences suggests also that the duplication event Z2d/Z2f predates diversification within catarrhines, but a larger repertoire of sequences is needed here to reconstruct phylogenetic relationships with confidence.

#### A3Z3 genes in primates

Orthologs of the A3Z3 gene can be found in the genomes of the catarrhines chimpanzee, bonobo, human, gorilla, Bornean and Sumatran orangutan, gibbon and macaque (Figure 1). The MRCA of these A3Z3 genes dates back to 31 Mya (95% HPD 26–34). In all these species the A3Z3 gene appears as a single copy, with no evidences of gene duplication. Additionally, the phylogenetic relationships among these genes and the inferred timing for the nodes perfectly match those of the corresponding species. The most parsimonious explanation for the absence of A3Z3 in platyrrhines and in other primates is therefore our lack of information about the locus, and it can be anticipated that the missing A3Z3 genes should be identified in the future, as genome coverage increases.

#### A3 genes in rodents

In the rodent *Cavia porcellus*, as in rat and mouse, the Z2Z3 genes are fused and the A3Z1 gene is missing. Considering the presence of A3Z1 in Primates, the last common ancestor of Muridae and Caviidae probably possessed already 50–85 Mya the genome organization found in the extant rat and mouse genomes. Precise answers on the timing for the A3Z1 deletion event and for the Z2Z3 fusion event will need to wait until new genomic information is available. Interesting sequences could come from the genomes of squirrels, which are basal to Muridae/Caviidae, or from rabbits, which constitute together with rodents the Glires taxon, sister to Primates.

### Evolution of the A3 loci in Laurasiatheria

#### A3 genes in cetartiodactyla

In the group of Cetartiodactyla (even-toed ungulates) the evolution of the individual A3 genes matches the evolution of the host genomes (Figure 1), although there is controversy about the phylogenetic relationships among mammalian orders here [46]. The genomes of cow and sheep present single copies of the A3Z1, A3Z2 and A3Z3 genes, with no evidences for gene duplications. In the A3 locus in the pig, the A3Z1 gene however seems to have been lost during evolution [15,47]. The presence of a A3Z1 gene in the genomes of cow and sheep suggests that the loss of the A3Z1 gene in pig genome occurred after the split that generated the Suidae lineage.

#### A3 genes in horses

**Figure 3:**
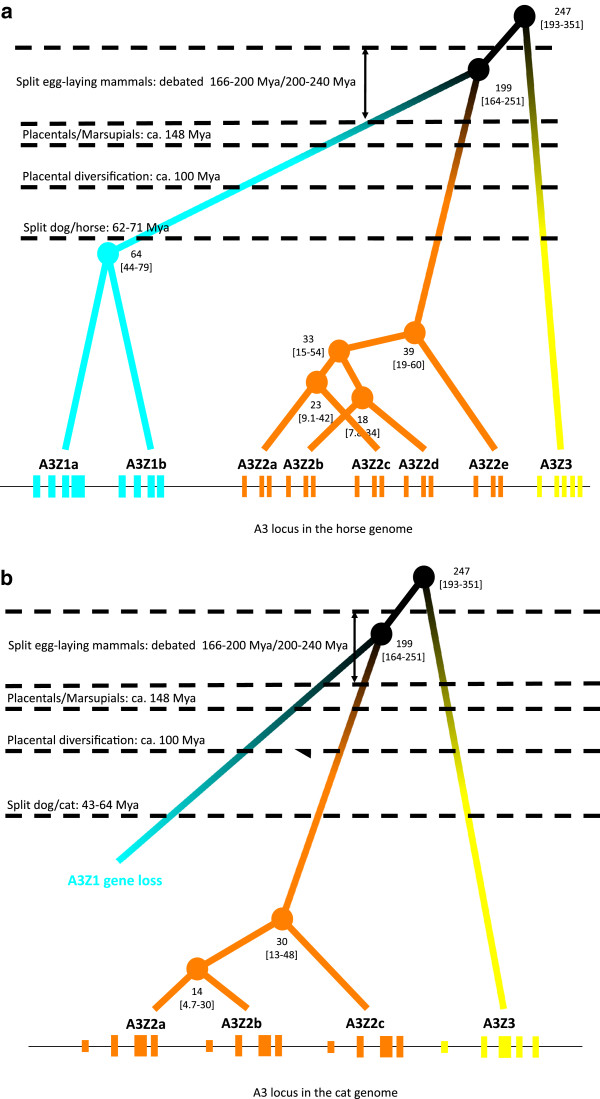
**Projection of the A3 phylogenetic relationships onto the horse (a) and cat (b) genomic loci.** Exon composition and gene arrangement of the A3 genes are displayed. Labels indicate median age [95% HPD] of the corresponding nodes. Several milestones in the evolution of mammals are indicated.

Modern horses have six A3 genes (Z1a, Z1b, Z2a, Z2b, Z2c, Z2d, Z2e, A3Z3) [27,48], which arose from the ancestral A3Z1, A3Z2 and A3Z3 genes after relatively recent duplication events (Figure 3a). The A3Z1 locus in the horse genome experienced a duplication event ca. 64 Mya (95% HPD 44–79), whereas the A3Z2 locus underwent three rounds of expansion between 39 and 18 Mya. The phylogenetic relationships as well as the accord in timing for the last two duplications suggest that the tandem of ancestral Z2ac and Z2bd genes underwent duplication in a single step, generating the present day arrangement Z2a Z2b Z2c Z2d Z2e [27]. The Equus genus dates back to ca. 4 Mya, posterior to the duplication events that shaped the A3 loci in horses. Thus, it is highly likely that other Equidae, such as zebra and donkey also present a horse-like A3 gene locus.

#### a3 genes in carnivora

Evidences of A3 genes can be found in the genome of canids and felids. The A3Z3 gene is present in all carnivores genomes analysed, but the A3Z1 gene is missing in Felidae and the A3Z2 gene is missing in Canidae (Figure 1,3b). Both gene loss events must have occurred independently after the split between cat and dog lineages, some 43–64 Mya. In the genome of the domestic cat (*F. catus*) we identified four A3 genes (Z2a, Z2b, Z2c, and Z3), while only one expressed A3Z2 and one A3Z3 gene could be found in diverse species in the Panthera genus [16]. The duplication events that generated the three A3Z2 genes in the genome of the cat date 30 Mya (95% HPD 13–48) and 14 Mya (95% HPD 4.7-30) (Figure 3b). Although fine details in the evolution of felids are a matter of debate, the MRCA of Pantherinae and Felinae could have lived some 12 Mya [49]. The fact that we have found the A3Z2 gene in lion, tiger and leopard –all of them Pantherinae- basal to the A3Z2 genes in cat, puma and lynx –all of them Felinae- suggests that the split between Pantherinae and Felinae predated the duplication events that generated the A3Z2 diversity in cats. Further, three different mRNA sequences were retrieved from lion and two from tiger [16]. If these mRNA sequences originated from different genes this would imply that independent gene duplications could have occurred in the Panthera lineage. However, considering again the potential for multiple alternative splicing in the A3 genes, the analysis of genomic sequences is required to determine whether panthers and cats have found similar, convergent evolutionary answers to a selective pressure that we still need to identify.

### Evidences for selection in the evolution of A3 loci

**Figure 4:**
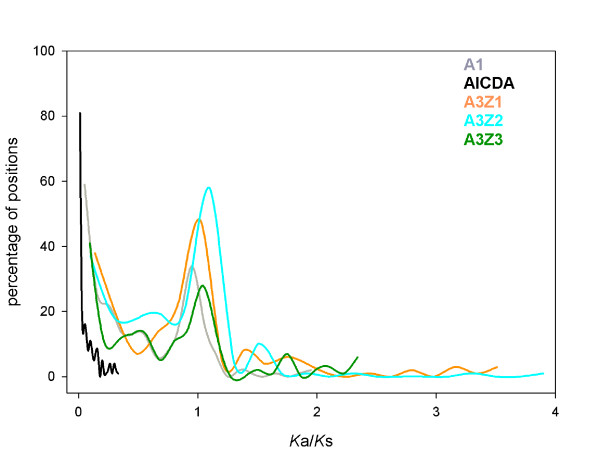
**Histogram showing the distribution of the percentages of *Ka/Ks *values for each position for the genes A1, AICDA, A3Z1, A3Z2 and A3Z3 (colour code in the inset).**

**Figure 5:**
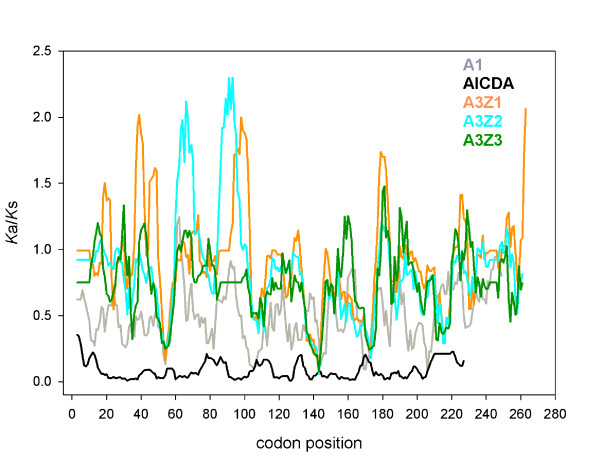
**Slide window analysis showing the variation of *Ka/Ks *along the corresponding sequence (colour code in the inset).** Values have been computed using a window of five positions and a step of one.

The A3 genes of primates, rodents, felids, horses and pigs have been described to be under a positive selection [15,16,50-53]. We have searched for hints of positive selection in the sequences in our dataset using Bayesian inference. The AICDA gene, the sister taxon of the A3s, showed to be under strict purifying selection while all A3Z1, A3Z2 and A3Z3 genes contained residues under positive selection (Table 1). Considering all sequences, the distribution of Ka/Ks values was clearly multimodal (Figure 4) for all three A3 genes, with around 25% of positions under strict purifying selection, with *Ka/Ks* values below 0.25, and above 10% of positions under positive selection, with *Ka/Ks* values above 1.1. To exclude that these results were driven by the sequences gathered from Primates the calculations were repeated after excluding them from the dataset, with a similar outcome (Table 1). A slide-window analysis showed that the *Ka/Ks* profiles were similar along the sequences of the three genes (Figure 5), reflecting that the repertoire of sites that are allowed to mutate and to explore sequence space are not evenly distributed and that to a certain extent localise to similar positions in the three A3Z1, A3Z2 and A3Z3 genes. Positions around codons 55 and 145 exhibit very low *Ka/Ks* values in all three A3 genes. There is a better coincidence of the *Ka/Ks* profiles in the C-terminus of the sequences analysed, while there is ample variation in the first 100 codons of the alignment, with A3Z1 and A3Z2 presenting islands of increased the *Ka/Ks* values that do not always overlap. The global distribution of *Ka/Ks* values in all sequences is depicted in Figure 4, where the complexity of the distribution is evident, showing the existence of different site levels of purifying selection, a large proportion of sites evolving close to neutrality and a 10-20% of the area below the curve corresponding to sites under diversifying selection (Table 1).

**Table 1:** **Statistics for the *Ka/Ks *values obtained either with the Mechanistic Empirical model (MEC) or with Random Effects Likelihood (REL) estimates**

		**All sequences**					**Excluding primates sequences**		
		**A1**	**AICDA**	**Z1**	**Z2**	**Z3**	**Z1**	**QZ2**	**Z3**
MEC ka/ks	quantil 25%	0.10	0.0067	0.29	0.26	0.19	0.44	0.26	0.15
	median	0.40	0.036	0.90	0.88	0.65	0.92	0.8	0.61
	quantil 90%	1.0	0.19	1.7	1.4	1.7	2.2	1.2	1.7
	maximum value	1.9	0.34	3.6	3.8	2.4	3.5	2.6	2.4
REL dN‒dS	percentage of sites under positive|puryfying selection	5.4|34	0|100	1.0|15	7.5|14	5.0|12	2.0|8.2	16|13	6.8|12

### Evolutionary rate has decreased in the three A3 subfamilies

**Figure 6:**
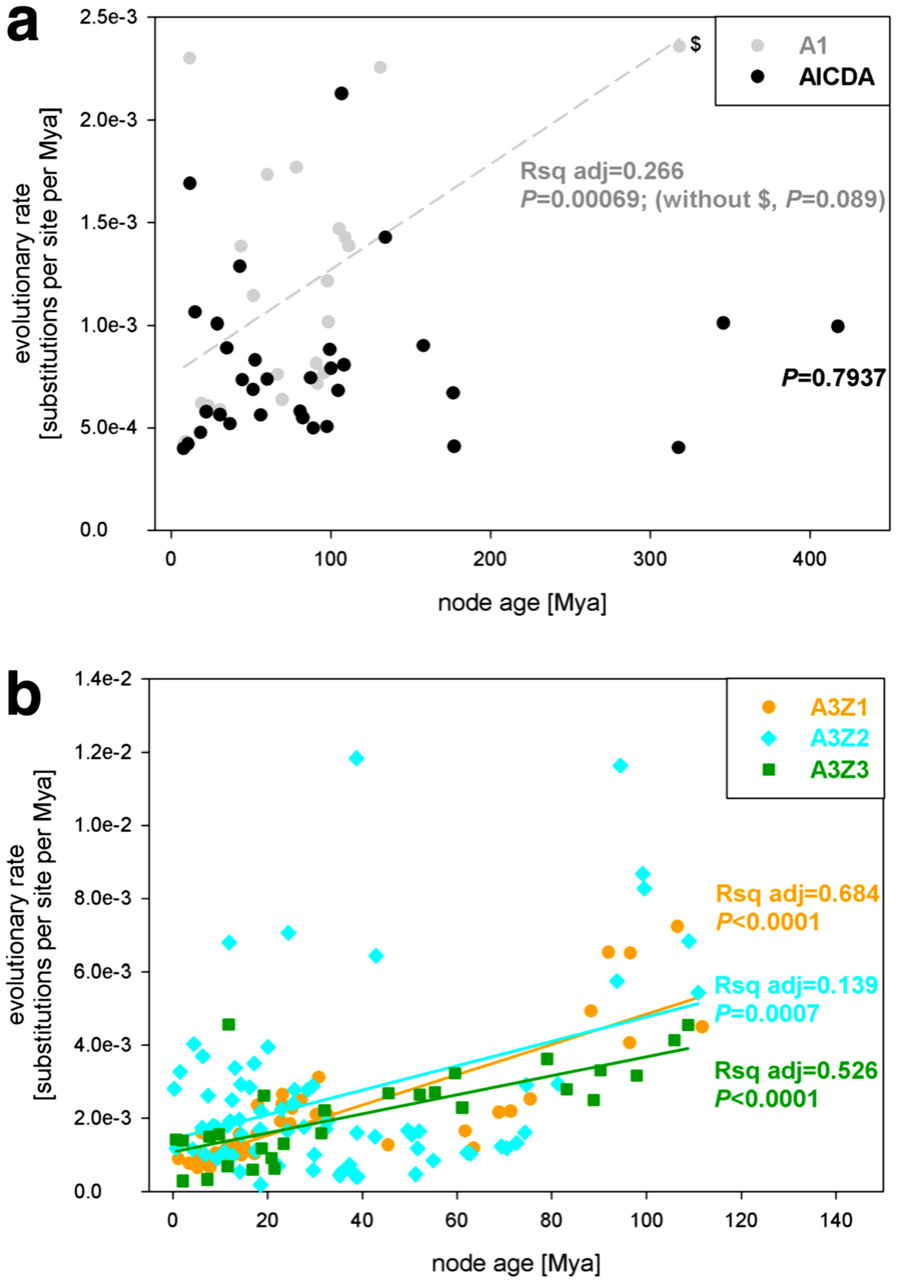
**Graph plotting the evolutionary rate against the node age for (a) A1 and AICDA sequences and (b) A3Z1, A3Z2 and A3Z3 sequences.** Colour code in the inset. Values for the goodness and significance of the fit to a straight line are provided. For the A1 genes, the values with and without the indicated point ($) are given. When this value is not included in the analysis the trend is not statistically significant.

The introduction of the variable time in the phylogenetic analyses allows to identify variations of the evolutionary rates in different taxa. The global mutation rate for our sequences set was 1.62·10^-3^ substitutions per site per My (given as Huber M-estimator, Huber-M; median absolute deviation, MAD, 1.07·10^-3^). The evolutionary rates were not homogeneous for the five gene families analysed (one-way ANOVA, *F* ratio = 7.414, *p* < 0.0001). Regarding the A1 genes the evolutionary rate is around 1.11 10^-3^ substitutions per site per My, (Huber-M, Table 2). For this gene family there has been a significant decrease in evolutionary rate with time (*P* = 0.00069, Figure 6a), but it is mainly lead by the values sampled for the oldest node at the root of the family. When this node is not included in the analyses, the evolutionary rate has not significantly varied with time in the last 150 My (*P* = 0.089, Figure 6a). The AICDA genes, the sister group to A3s, displayed the lowest evolutionary rate among the sequences studied (Huber-M 7.41·10^-4^ substitutions per site per My) and this value has not experienced changes with time in the last 400 My (Figure 6a). All A3s however displayed increased evolutionary rate values, around 2.5 times higher than the AICDA sistergroup (Table 2). Very interestingly, the evolutionary rate for each of the three A3 subfamilies has significantly decreased in the last 100 My. For A3Z1 and A3Z3, the linear dependence with time explains a large variation in the evolutionary rate, above 50% for both genes (Figure 6b). In the case of the A3Z2 this dependence is still significant but less obvious, and explains less than 15% of the total variability in evolutionary rate.

**Table 2:** Evolutionary rates for the different large clades described measured as substitutions per site per million of years, as inferred from the Bayesian time analyses

	**Evolutionary rate (substitutions per site per My)**				**Evolution of the evolutionary rate (Δ evolutionary rate per My)**	
	**Huber M‒estimator**	**Median absolute deviation**	**Mean**	**95% confidence interval of the mean**	**Slope**	**Standard error**
all	1.62 10^-3^	1.07 10^-3^	1.97 10^-3^	4.31 10^-4^ – 6.44 10^-3^	NA	NA
A1	1.11 10^-3^ A,B	5.86 10^-4^	1.15 10^-3^	4.49 10^-4^ – 2.29 10^-3^	5.15 10^-6^	1.7 10^-6^
AICDA	7.41 10^-4^ A	2.74 10^-4^	7.99 10^-4^	4.06 10^-4^ – 1.53 10^-3^	NS	NS
A3Z1	1.79 10^-3^ B,C	9.88 10^-4^	2.14 10^-3^	7.49 10^-4^ – 6.50 10^-3^	4.15 10^-5^	4.4 10^-6^
A3Z2	2.06 10^-3^ C	1.28 10^-3^	2.60 10^-3^	4.52 10^-4^ – 7.61 10^-3^	3.34 10^-5^	9.4 10^-6^
A3Z3	2.13 10^-3^ C	1.38 10^-3^	2.15 10^-3^	4.17 10‒4 – 4.40 10^-3^	2.61 10^-5^	4.7 10^-6^

Evolution of the evolutionary rate measured as variation of the substitution per site per (million of years)^2^. *NA* not analysed. *NS* correlation was not significant. Values connected by the same letter are not significantly different after a Tukey-Kramer honestly significant difference test.

## Discussion

A3 genes belong to a large superfamily of deaminases that edit nucleic acids and constitute the sister taxa to AICDA, as previous studies suggested [37,54]. Certain members of the deaminase family, such as A1, target RNA as a substrate, but it has been proposed that the ancestral activity may have been to target DNA [54]. Our results show that the MRCA of AICDA and A3, ca 420 Mya, predates the split between the lineages of zebra fish and humans. The subsequent duplication events of the ancestral A3 gene to generate the three extant A3Z1, A3Z2 and A3Z3 genes could not be dated with precision and overlap largely. The first one occurred ca. 247 Mya (95% HPD 193–351 Mya) and the second one ca. 199 Mya (95% HPD 164–251). Representatives of Laurasiatheria, Euarchontoglires and Afrotheria are found in all A3Z1, A3Z2 and A3Z3 subtrees. For Afrotherians, a few sequences were found basal to the A3Z1 subgroup (only sequences from the African bush elephant *Loxodonta Africana*), to the A3Z2 subgroup (the N-terminus from *L. Africana*) or to the A3Z3 subgroup (an *in silico* prediction from genomic DNA from the rock hyrax *Procavia capensis* and the C-terminus from one *L. africana* sequence). On the view of the evolutionary relationships and time divergences, we can conclude that the duplication events that originated the three ancestral A3 loci had already occurred well before diversification within placental mammals, which took place at some point between 95–120 Mya [55]. The presence of A3Z1 and A3Z3 representatives in Afrotheria and of A3Z1, A3Z2 and A3Z3 in Boreoeutheria sustains also this view. The estimated appearance of the most recent common ancestor of all A3s predates the split between placental mammals and marsupials 125–150 Mya, and also the split of monotremes at the base of the crown clade of modern mammals, debated between 160–200 Mya [56], and 203–238 Mya [57]. Thus, our data support an evolutionary scenario where the genome of the MRCA of all mammals already encoded for at least an ancestral A3, and where the genome of the MRCA of all placental mammals already encoded for a A3Z1-A3Z2-A3Z3 arrangement. This genomic arrangement is conserved in all placental mammals, with the A3 genes flanked by the CBX6 and CBX7 genes, as in chromosome 22 q13.1 in humans. This conserved chromosomic location could be used for targeting and sequencing of the A3 locus of selected species in the future. Unfortunately, the sequence gap between CBX6 and CBX7 is not resolved with certainty in the opossum and in the platypus genomes, and we cannot confirm whether the last common ancestor of Eutheria and Metatheria already possessed a single ancestral A3, the intermediate stage with two loci, or already three loci, as the ancestor of all placental mammals. A note of caution should be nevertheless stated here, since our results cannot go beyond the sequence dataset that we have been able to gather. In a number of genomes we have not been able to recover any relative of the A3 sequences. This holds true for Monotremes, Marsupials and Xenarthra. For other organisms we have not been able to detect certain A3 genes, such as the A3Z1 gene in the cat genome and in Rodentia, or the A3Z2 gene in the dog genome. Since for humans the existence of copy number variation in the A3A and A3B genes is documented in a good number of cases (Additional file 4: Figure S3), a similar situation can be detected in the future in other species. It is important to note here that obtaining genomic sequences of the A3 locus is not trivial, as it evolves under strong selective pressures [45]. In our current analyses we have opted for the most parsimonious explanation: e.g. the presence of an A3Z2 gene in Euarchontoglires and in Felidae and the absence in Canidae has been interpreted as the Boroeutherian ancestor carrying a copy of the ancestral A3Z2 gene, which may had got lost in the Canidae lineage. The hypothetical finding in the future of an A3Z2 gene in Canidae would not affect our timing results as all calibrations have been chosen on subtrees whose structure matched that of the corresponding species (Additional file 5: Table S2).

The results here presented show that the A3 loci in mammalian genomes are extremely variable and have undergone independent events of gene duplication followed by fixation, gene loss and gene fusion. The outcome of these events has lead to parallel evolution, as is the case of the loss of A3Z1 gene in rodents, dogs and pigs. The most striking result, however, is that the amplification of A3 genes and the fixation of duplicated genes has been selected and expanded in the population, independently in several taxa, such as the A3Z2 gene in Primates (Figure 2), horses (Figure 3a) and cats (Figure 3b) and the A3Z1 gene in Primates and horses. Further, an increase in the frequency of duplication events in the last 50 Mya can be observed in these three lineages, i.e. human, horse, cat (labelled with star symbols in Figure 1). Gene duplications have occurred in both A3Z1 and A3Z2, but not in the A3Z3 clade. Duplication of the A3Z3 gene seems thus to be unfavoured, possibly reflecting the trade-off between the advantages of an increased antiviral repertoire and the increased self-toxicity by gene dosage effect [18]. Also pointing in this direction, there is evidence for independent events of A3Z3 activity loss in human alleles [58]. Finally, further data that exemplify the broad variability in the A3 locus come from copy number variation studies in humans, with several reports on the loss of the A3A (A3Z1a) and/or the A3B (A3Z2a-A3Z1b) genes (Additional file 4: Figure S3).

Different taxa have selected similar solutions through independent events in the A3 locus in terms of gene duplications and/or deletions. Such parallel evolution may be the response to similar environmental changes, e.g. increased retroviral activity either exogenous or endogenous, which could account for the parallel expansion of the A3 family in different mammalian clades. The fixation of similar duplication events in different lineages suggests that the preservation of the duplicated genes has a positive effect in fitness. Further, the main activity of all A3 genes seems to be ssDNA editing, and the differences among them are rather related to substrate specificity [59,60], expression pattern [61,62], and virus specificity [2]. The fate of new A3 copies may thus be sub-functionalisation by broadening the spectrum of molecular targets that can be edited by the A3 proteins, and that this fine tuning of the edited DNA substrates results in an increase in fitness. Such shift in substrate specificity is also supported by the evidence of positive selection in A3s in primates, rodents, felids, horses [16,50-53,63], and our results confirm as well the presence of residues under positive selection in all A3Z1, A3Z2 and A3Z3 clades (Table 1). However, the antiviral relevance of the differential deaminase activity as a function of the ssDNA sequence still needs to be explored.

Our results show that residues evolving under neutrality co-occur with residues under positive and under purifying selection in the A1 and in the A3 genes, and that this holds true for all sequences considered together even after accounting for the excess of Primates sequences (Table 1 and Figure 4). For all these genes, there is a clear peak and a substantial proportion of sites evolving close to neutrality. Additionally, a substantial amount of area below the curve is located in the two tails of the multimodal distribution, corresponding to positions evolving under strict purifying selection and intermediate values of 0.5 < *Ka/Ks* < 0.8, and under positive selection. Previous descriptions had rather focused on specific genes in specific taxa, i.e. primates, rodents, horses, felids and pigs [16,50-53,63]. Our analyses have addressed the three A3 gene clusters in all mammals and show that multiple evolutionary pressures coexist in the same gene sequence. Similar cases of positive selection observed as an increase of *Ka/Ks* during subfunctionalisation of duplicated genes has been also documented in other gene families [64]. The biological interpretation of the significance of individual residues under positive selection needs to be analysed in the context of the evolution of the gene in which they reside and in the biochemical context of the exon combination present in the actually expressed proteins. Methods for detecting positive selection based on *Ka/Ks* ratios may result in false positives, as a relaxation of evolutionary constraints could also lead to an increase in this parameter [65]. Such constraint relaxation is specially expected to occur in genes that have undergone duplication events, as it is the case in the A3 genes. Thus, the actual meaning of sites under positive selection in the three A3Z1, A3Z2 and A3Z3 clades requires experimental confirmation. Additionally, gene duplication could be the result of the genomic arms race between target viruses and their hosts, as novel A3 proteins can counteract anti-antiviral activity raised in a virus that evolves anti-A3 mechanisms, such as the Vif or the Bet protein [19-22,24]. This combination could correspond to certain models among those proposed by Innan and Kondrashov [40] to classify the evolution of duplicated genes, such as modified duplication or diversifying selection. The evolution of the A3Z2 locus in primates, horse and cat, with several rounds of successive amplification events, suggests however that gene duplication may have occurred here through an adaptive radiation model, as proposed by Francino [66]. The new A3 copies could explore the sequence space that expands the family of substrates for the action of deaminases, typically by modifying the sequence context of the C- > U edition. Similarity among duplicated genes could also allow for gene conversion and/or recombination [67], further enlarging the repertoire of substrates. In analogy to gene conversion at the DNA level, similarity in DNA sequence between tandem duplicated genes may be responsible for the read-through mRNA species that encompass exons from different genes, and that have been described in cats [16,68], horses [27] and pigs [15,47] and have also been communicated in humans [69] (Additional file 6: Figure S4). Currently more than 500 ESTs in the databases map onto the A3 human loci, with a number of them containing spliced exons from different A3 genes (Additional file 6: Figure S4). This additional generation of diversity adds to the high number of alternatively spliced mRNAs originated from each individual A3 gene, especially of those composed of two fused “mono” A3s, either A3Z2-A3Z1 or A3Z2-A3Z2. In these cases we face fused genes that retain their individual coding capacity, can generate multiple splice alternatives and show potential to encode for read-through mRNAs. Finally, an additional level of spatiotemporal complexity to the regulation of this antiviral activity arises from A3 expression heterogeneity linked to cell type, tissue, developmental time or exposure to foreign DNA.

The architecture of several tandem copies of paralogs in a genome facilitates non-homologous recombination among paralog copies resulting in gene conversion, as has been suggested for the A3G gene in humans [67]. A high degree of gene conversion is expected to result in an increased degree of sequence similarity through concerted evolution [70,71], with the undesired outcome of rendering younger divergence times [72]. Our dataset is unfortunately not suited for an in-depth analysis of gene conversion, since it includes well-characterised mRNAs, but also ESTs that may contain exons that originate from different genes and putative mRNAs inferred from genomic sequences. A proper analysis will need good quality genomic sequences with enough sampling of individuals if variations found in the human A3 locus in terms of gene copy number and indels (Additional file 4: Figure S3 and Additional file 6: S4) appear also in other species. Such analyses will need to address gene order within the locus, but also exon-intron order within the genes, and alternative splicing and mRNA read-through. Nevertheless, certain cases can be considered. The confounding role of gene conversion may have disturbed the phylogenetic reconstructions for the A3Z2 genes. In Primates, the A3Z2a, A3Z2c and A3Z2e A3Z2g paralogs have appeared after two duplication events from their common ancestor around 73 Mya (Figure 2). The topology for the A3Z2g subtree matches well the phylogeny of the corresponding species (Additional file 2: Figure S1), and gene conversion may have had a limited impact here. The evolution of the A3Z2a, A3Z2c and A3Z2e genes however cannot be reconstructed with confidence, and appears as a series of small branches with small support values, which could be interpreted as a signature of gene conversion. The same holds true for the rest of the A3Z2 paralogs, A3Z2b/d/f. Finally, a second candidate for gene conversion to have occurred is the A3Z2a/b gene tandem in the cat genome [16].

The AICDA genes show the lowest evolutionary rate among the five clades studied, 1.11 10^-3^ substitutions per site per My, significantly different from the values inferred for the three A3 genes, which are around three times higher (Table 2). Very interestingly, we have found that these evolutionary rates have decreased for the A3 genes in the last 100 My, whereas for the AICDA and the A1 genes there are no significant variations in the evolutionary rate with time (Table 2). We interpret the different outcome for the A3 genes and for their sister taxa as an evidence for our results being genuine rather than an artefact from the evolutionary inference. The simultaneous identification of positions evolving under positive selection in the three A3 genes and the finding of a trend towards decrease with time of the evolutionary rate in the same genes are not contradictory. Our calculations for *Ka/Ks* have been performed separately for each position, while the values inferred for the evolutionary rates refer to the corresponding nodes in the phylogenetic reconstruction. In our scenario of gene duplication and gene family expansion, we interpret that the episodes of gene duplication have lowered the restrictions on the duplicated copies of the genes, thus allowing for increased evolutionary rates. Subfunctionalisation of the sister copies may have yielded one population of conserved sites evolving under purifying pressures (e.g. those sites that are indispensable for the deaminase activity), one population of sites positively selected sites (e.g. those that are responsible for the differential substrate recognition) and a third large majority of sites evolving close to neutrality. The decrease in evolutionary rate possibly reflects a plateau in the fixation of the novel function. In the A3Z2 genes, which have undergone the largest number of duplication events, the large variation in evolutionary rate for the different nodes (Table 2) and the lower proportion of the decrease in evolutionary rate that is explained by the independent variable time alone (less than 15%; Figure 6b) supports further the idea that gene duplication fosters a transient increase in evolutionary rate.

We have not been able to identify in the databases sequences that could be orthologs of the A3 genes in Marsupials or in Monotremes. We have dated the MRCA of the A3 genes around 246 Mya (95% HPD 192–351 Mya), and the second split that generated the MRCA of A3Z1 and A3Z2 around 199 Mya (95% HPD 164–250). The timing for the crown clade of mammals (i.e. the split between Monotremata and Theria) as well as the timing for the split between marsupials and placentals are controversial, ranging between 160 and 240 Mya [55-57]. Three explanations could thence account for the absence of extant A3 genes in monotremes and marsupials: i) we simply lack information and further sequencing will provide us with the missing genes; ii) members of one or of both groups may indeed have lost the A3 genes; and iii) genes may be exclusive to placentals if the MRCA of placental mammals predates the appearance of the MRCA of all A3s. Our interpretation implies in any case that the genome of the placental ancestor already encoded for an A3 locus with the arrangement A3Z1-A3Z2-A3Z3. The absence of A3 genes in Xenarthra, the fourth large clade within placental mammals, must therefore imply either gene loss or incomplete coverage of the A3 locus in the two species analysed. Since we have described that loss of certain A3 genes has occurred in parallel in different lineages, as in Rodents and in Artyodactyla, it is conceivable that in certain lineages the loss of all A3 genes may have been selected. The adaptive value of an enlarged armoury against viruses is obvious, and evidences supporting positive selection of the A3 genes in different branches of the mammalian tree are strong. The intriguing hypothesis of the total local loss of A3 genes might imply that the constraints and pressures imposed by viral infections can largely vary among different taxa. Certain host lineages may thus either be less exposed to (certain) viruses, and/or may have evolved alternative antiviral strategies. An exciting question that arises from our dating results is the relative coincidence in time between speciation events and gene duplication events, as exemplified in Figures 2 and 3. It could be speculated that gene duplication of the A3 genes may trigger speciation, possibly through the differential fitness against viral infections that the additional A3 gene copy provides. Further genetic and functional research will be required to elucidate the fitness landscapes integrating viral pressures, expansion of the A3 repertoire and concomitant risks for the own genetic information.

## Conclusions

The A3 gene family appeared together with the ancestral mammals. Independently in certain lineages, the A3 locus was expanded through a series of tandem duplications, best exemplified in Primates. The repertoire of A3 proteins is additionally expanded through splice alternatives and read-through mechanisms, resulting in broader substrate specificity and finer regulation of DNA modification potential. We have shown that this diversity has been generated by series of tandem duplication in the A3 locus probably followed by positive selection and/or relaxation of constraints and resulting in sub/neo-functionalisation. Such evolutionary solution has been independently selected in several lineages: Primates, felids and equids. Our findings constitute a paradigm of genomic parallel evolutionary solutions in the framework of the arms race between viruses and their hosts.

## Methods

### Dataset

Annotated sequences were retrieved from GenBank and Ensembl Genome Browser, the data set was completed with cDNA sequences and with genomic sequences putatively encoding for products similar to A3 after iterative BLAT, tBLASTn and PSI-BLAST searches. Our final dataset included sequences from within Laurasiatheria (seven Carnivora, seven Cetartiodactyla, one Perissodactyla and one Chiroptera species), Euarchontoglires (three Rodentia and seventeen Primates species) and Afrotheria (one Hyracoidea and one Proboscidea species). Fused genes as in human A3Z2a-A3Z1b were split and analysed as two sequences.

A selection of 24 APOBEC1 (A1) sequences (21 Eutheria species, two Metatheria and one Aves species) and 34 Activation induced cytidine deaminases (AICDA) sequences (23 Eutheria species, three Metatheria, one Prototheria, two Aves, two Amphibia and three Actinopterygii species) and were used as outgroups. The final sequence set comprised 202 sequences. A list of species and accession numbers is given in Additional file 1: Table S1.

### Phylogenetic inference

Sequences were aligned with mafft ( http://mafft.cbrc.jp/alignment/software/) at the amino acid level and back-translated into nucleotides for phylogenetic analyses. This matrix is available as Additional file 7 and from IGB on request. Maximum likelihood (ML) inference was performed with raxml_v7.2.8 ( http://www.exelixis-lab.org/) [73] using 5000 bootstrap cycles with the GTR + G4 model and introducing three partitions, one per codon position (number of patterns 290, 290 and 298, respectively) and allowing for different total tree length for each partition. Bayesian inference was performed with phylobayes v3.3 ( http://www.phylobayes.org) [74] using the same dataset, under the GTR model, removing constant sites, running two independent chains and checking for convergence comparing discrepancies among partitions. Trees obtained after ML and Bayesian reconstructions were compared regarding topological congruence as well as pairwise distances using K-TreeDist [75], with the following result: Robinson-Foulds distance, 67/401 (16.7%); K-score, 1.07; scale factor, 0.542. Both ML and Bayesian topologies rendered A3Z1, A3Z2 and A3Z3 as monophyletic, and A1 as the outgroup to A3s and AICDA. Twenty-five local topologies, highly supported in both analyses (above 90% bootstrap support and above 0.98 Bayesian posterior probability) and consistent with the phylogenetic relationships of the hosts were identified and used for molecular dating, using truncated uniform priors based on fossil calibration dates, as suggested by Benton and Donaghue [56,76]. Calibration dates are listed in Additional file 5: Table S2. All discussion on the results obtained refers to the dates and analyses proposed by TimeTree ( http://www.timetree.org/index.php) [77]. The backbone tree for time inference was constructed with RAxML v7.2.8 with the same settings as described above, further forcing monophyly for each of the nodes used for calibration (−*g* option), plus for the sequences belonging to the crowngroups Afrotheria, Laurasiatheria and Euarchontoglires within each of the clades A1, AICDA, A3Z1, A3Z2 and A3Z3, respectively. This tree was not significantly worse than the original one, under the maximum-likelihood framework, as evaluated with a Shimodaira-Hasegawa test [78] implemented in RAxML v7.2.8. The comparison between the unconstrained and the constrained maximum likelihood trees rendered the following values: Robinson-Foulds distance, 71/401 (17.7%); K-score; 0.780; scale factor, 0.823. Using this tree, Bayesian time inference was performed with phylobayes v3.3 using a discrete gamma distribution with eight categories under the GTR matrix of exchange rates, using a log-normal autocorrelated relaxed clock together with a uniform prior on divergence times. A gamma prior of mean 550 and standard deviation 200 Mya was specified for the age of the root. The results from three 50-million steps independent chains were combined and analysed.

### Positive selection analysis

Evidence for positive selection was analysed in a Bayesian framework using selecton v2.4 ( http://selecton.tau.ac.il/) and datamonkey ( http://www.datamonkey.org/). The alignments for the A1, AICDA, A3Z1, A3Z2and A3Z3 genes were analysed separately for the whole sequence set, and those for A3Z1, A3Z2 and A3Z3 were additionally analysed after excluding the Primates sequences, to discard that the results were driven by the behaviour of sequences from this clade. With selectonv2.4, the Mechanistic Empirical Model (MEC) was tested against the M8a model [79]. In both cases the topology of the best-known maximum likelihood tree previously obtained was used as scaffold for the calculation of synonymous *Ks* and non-synonymous *Ka* mutations. Briefly, MEC expands an empirical amino acid replacement matrix –in our case the WAG matrix [80], the best scoring one as determined by ProtTest [81] among those available in the selecton algorithm- into a codon replacement matrix. This way, the chemical similarity between amino acids is incorporated when calculating non-synonymous substitutions [79]. The likelihood of this model was tested against the M8a model, which does not allow for positive selection, using the Akaike information criterion [82]. For all of the sequence sets the MEC model was preferred over the M8a. For each position, the value for Ka/Ks was calculated. The variation of *Ka/Ks* along the corresponding sequence was analysed using a slide-window analysis of width five and step one. With datamonkey, the Random Effects Likelihood (REL) method was applied, which assumes an underlying nucleotide substitution model and allows for rate variation in both *Ka* and *Ks* substitution rates [83]. All analyses were repeated after excluding the Primates sequences from the dataset, in order to exclude putative biases inherent to the overrepresentation of this clade in terms of sequence composition and presence of the different genes in the genome. Statistic analyses were performed with R v1.40 and with JMP v7.0.2.

## Competing interests

The authors declare that they have no competing interests.

## Authors’ contribution

Study conception: CM and IGB. Data collection and analysis: AW and IGB. Data interpretation: IGB. Manuscript draft: CM, AW and IGB. All authors read and approved the final manuscript.

## Supplementary Material

Additional file 1 Table S1.Taxonomy and sequence accession numbers of the sequences used in this study. Click here for file

Additional file 2 Figure S1.Best‒known maximum likelihood tree for the A3 genes analysed, AICDA and for the outgroup A1. Colour code describes mammalian taxa, as in Figure 1. Values in the nodes depict bootstrap support. Click here for file

Additional file 3 Figure S2.mRNAs deposited in the databases originating from the human A3 locus, after the USCS Genome Browser ( http://genome.ucsc.edu/cgi‒bin/hgTracks), showing human chromosome 22, positions 39,250,000 to 39,550,000, accessed on December 13th 2011. Click here for file

Additional file 4 Figure S3.Bayesian dated tree for the A3 genes analysed, AICDA and for the outgroup A1. Bars around the nodes describe the 95% HPD for the inference of the node age. Click here for file

Additional file 5 Table S2.Values for calibration used in this study. Click here for file

Additional file 6 Figure S4.Copy number variation in the human A3 locus, after the Database of Genomic Variants ( http://projects.tcag.ca/cgi‒bin/variation/gbrowse/hg19/), showing human chromosome 22, positions 39,250,000 to 39,550,000, accessed on December 13th 2011. Click here for file

Additional file 7 Final sequence matrix, codon-aligned. Click here for file
