# Supplementary figures and images for: An ancient history of gene duplications, fusions and losses in the evolution of APOBEC3 mutators in mammals
Source: BMC Evol Biol. 2012 May 28;12:71. doi: 10.1186/1471-2148-12-71 (PMC3495650; doi:10.1186/1471-2148-12-71)

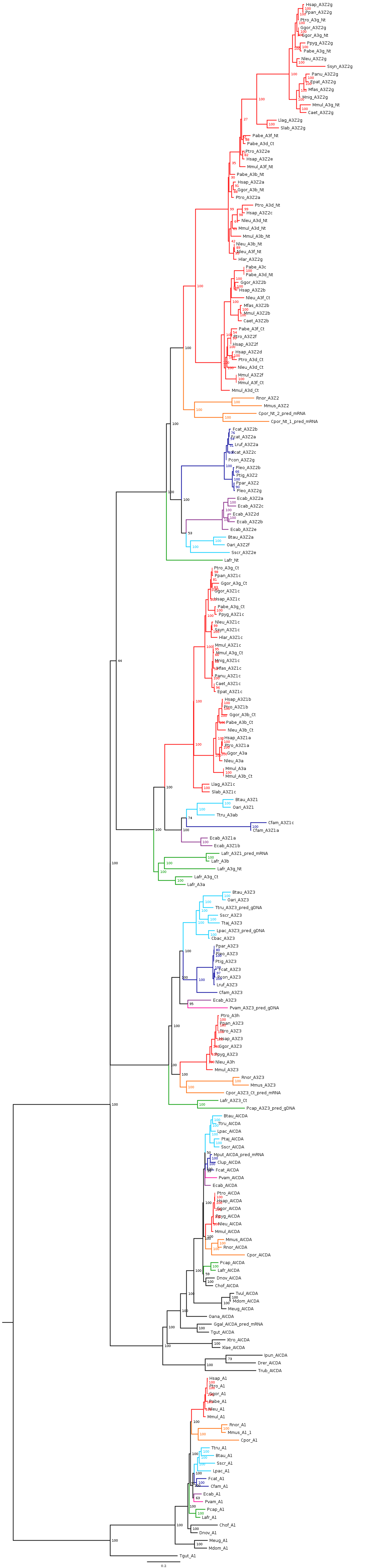

Supplement: Additional file 2 — Figure S1.Best‒known maximum likelihood tree for the A3 genes analysed, AICDA and for the outgroup A1. Colour code describes mammalian taxa, as in Figure 1. Values in the nodes depict bootstrap support. [file 1471-2148-12-71-S2.png]

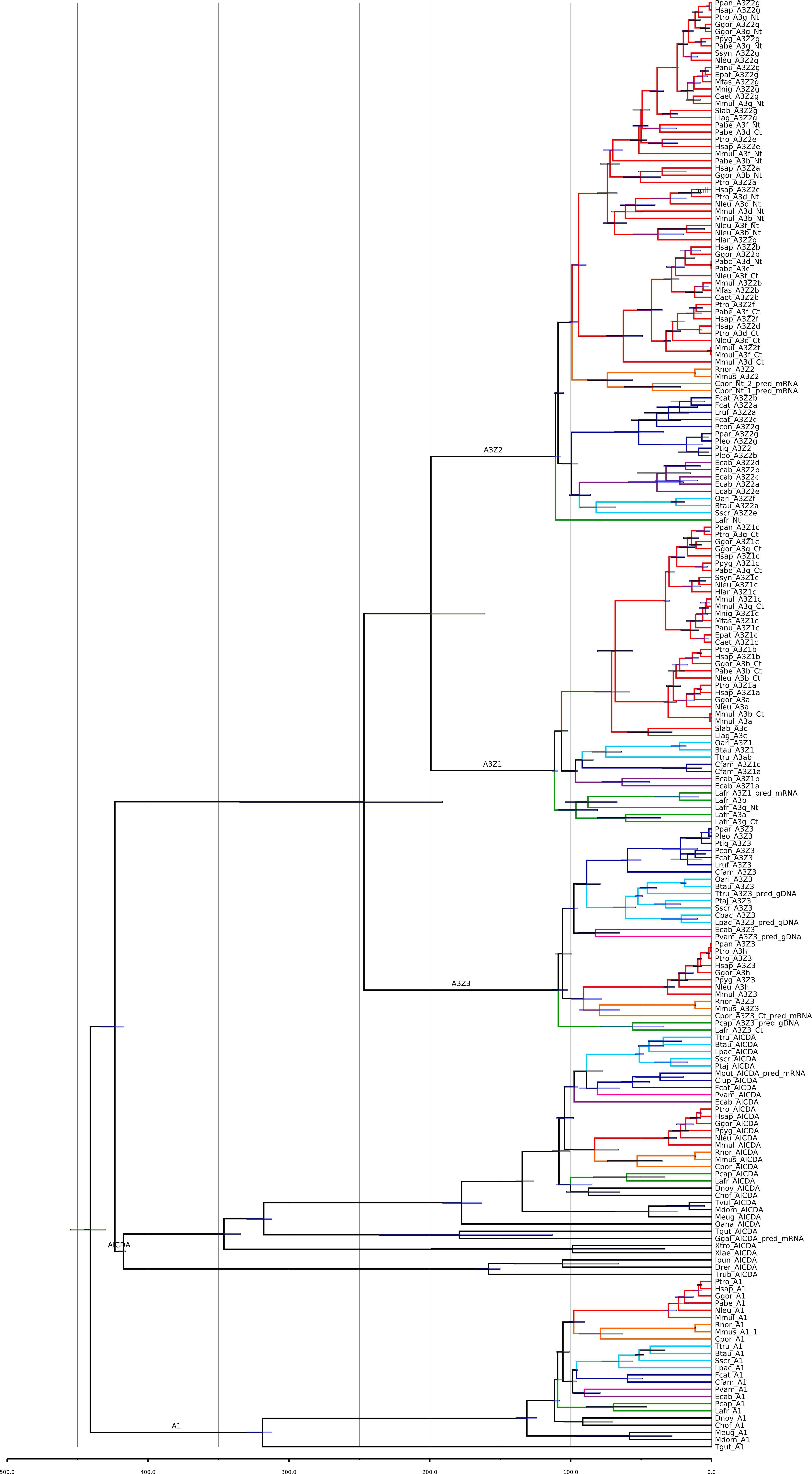

Supplement: Additional file 3 — Figure S2.mRNAs deposited in the databases originating from the human A3 locus, after the USCS Genome Browser ( http://genome.ucsc.edu/cgi‒bin/hgTracks), showing human chromosome 22, positions 39,250,000 to 39,550,000, accessed on December 13th 2011. [file 1471-2148-12-71-S3.png]

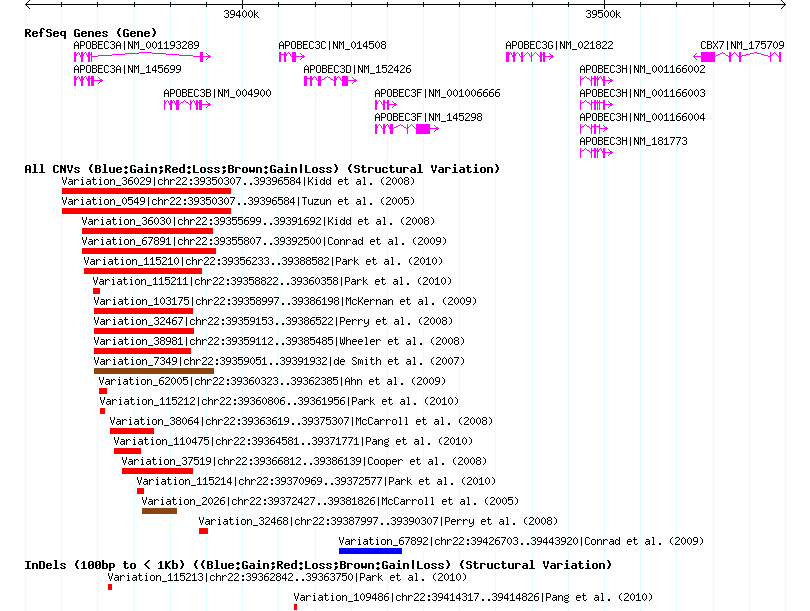

Supplement: Additional file 4 — Figure S3.Bayesian dated tree for the A3 genes analysed, AICDA and for the outgroup A1. Bars around the nodes describe the 95% HPD for the inference of the node age. [file 1471-2148-12-71-S4.png]

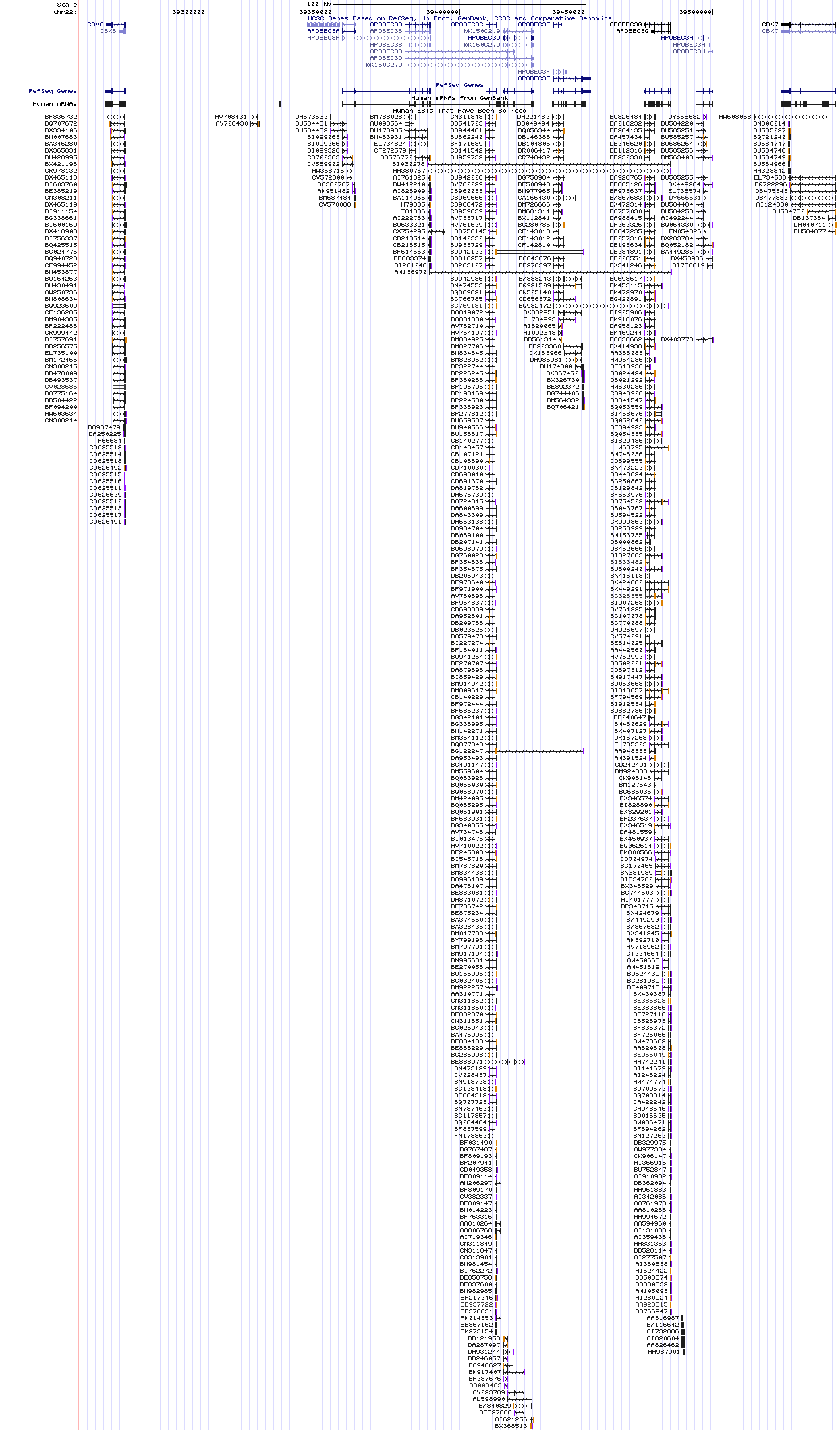

Supplement: Additional file 6 — Figure S4.Copy number variation in the human A3 locus, after the Database of Genomic Variants ( http://projects.tcag.ca/cgi‒bin/variation/gbrowse/hg19/), showing human chromosome 22, positions 39,250,000 to 39,550,000, accessed on December 13th 2011. [file 1471-2148-12-71-S6.png]
